# Predominant membrane localization is an essential feature of the bacterial signal recognition particle receptor

**DOI:** 10.1186/1741-7007-7-76

**Published:** 2009-11-13

**Authors:** Miryana Mircheva, Diana Boy, Benjamin Weiche, Friederike Hucke, Peter Graumann, Hans-Georg Koch

**Affiliations:** 1 Institut für Biochemie und Molekularbiologie, ZBMZ, Universität Freiburg, Stefan-Meier-Str. 17, 79104 Freiburg, Germany; 2 Mikrobiologie, Fakultät für Biologie, Universität Freiburg, Schänzlestraße 1, 79104 Freiburg, Germany

## Abstract

**Background:**

The signal recognition particle (SRP) receptor plays a vital role in co-translational protein targeting, because it connects the soluble SRP-ribosome-nascent chain complex (SRP-RNCs) to the membrane bound Sec translocon. The eukaryotic SRP receptor (SR) is a heterodimeric protein complex, consisting of two unrelated GTPases. The SR*β *subunit is an integral membrane protein, which tethers the SRP-interacting SR*α *subunit permanently to the endoplasmic reticulum membrane. The prokaryotic SR lacks the SR*β *subunit and consists of only the SR*α *homologue FtsY. Strikingly, although FtsY requires membrane contact for functionality, cell fractionation studies have localized FtsY predominantly to the cytosolic fraction of *Escherichia coli*. So far, the exact function of the soluble SR in *E. coli *is unknown, but it has been suggested that, in contrast to eukaryotes, the prokaryotic SR might bind SRP-RNCs already in the cytosol and only then initiates membrane targeting.

**Results:**

In the current study we have determined the contribution of soluble FtsY to co-translational targeting *in vitro *and have re-analysed the localization of FtsY *in vivo *by fluorescence microscopy. Our data show that FtsY can bind to SRP-ribosome nascent chains (RNCs) in the absence of membranes. However, these soluble FtsY-SRP-RNC complexes are not efficiently targeted to the membrane. In contrast, we observed effective targeting of SRP-RNCs to membrane-bond FtsY. These data show that soluble FtsY does not contribute significantly to cotranslational targeting in *E. coli*. In agreement with this observation, our *in vivo *analyses of FtsY localization in bacterial cells by fluorescence microscopy revealed that the vast majority of FtsY was localized to the inner membrane and that soluble FtsY constituted only a negligible species *in vivo*.

**Conclusion:**

The exact function of the SRP receptor (SR) in bacteria has so far been enigmatic. Our data show that the bacterial SR is almost exclusively membrane-bound *in vivo*, indicating that the presence of a soluble SR is probably an artefact of cell fractionation. Thus, co-translational targeting in bacteria does not involve the formation of a soluble SR-signal recognition particle (SRP)-ribosome nascent chain (RNC) intermediate but requires membrane contact of FtsY for efficient SRP-RNC recruitment.

## Background

The signal recognition particle (SRP) dependent targeting constitutes a universally conserved protein targeting pathway that ensures the co-translational delivery of substrates to the membrane-bound Sec translocon [1]. Co-translational targeting is achieved because SRP recognizes its cargo early in translation [2,3]. Subsequently, the SRP-ribosome nascent chain complex (SRP-RNC) is targeted to the membrane-bound SRP receptor (SR) [4,5], which in eukaryotes is composed of two GTPase subunits. SR*α *is tethered to the ER membrane via the membrane integral SR*β *subunit and is responsible for binding the SRP-RNC. However, SR*β *is not only required for the anchoring of SR*α *to the membrane, it is also involved in coordinating the transfer of the RNC to the Sec translocon [6,7].

Despite its conservation, the SRP-dependent targeting in bacteria such as *Escherichia coli *deviates from the eukaryotic pathway because, here, SRP delivers almost exclusively inner membrane proteins, while secretory proteins are targeted by the post-translational SecA/SecB pathway [1]. In addition, the bacterial SR consists of only the SR*α *homologue FtsY, but lacks a membrane-integral SR*β *subunit. However, FtsY can bind to the *E. coli *membrane via two lipid-binding helices, one located at the N-terminus of FtsY and a second at the interface between the non-essential A-domain and the essential N-domain [8,9]. Deleting both lipid-binding helices prevents targeting [9-11], which supports data showing that only membrane-bound FtsY is able to induce efficient dissociation of SRP from the RNC [12]. FtsY also contacts the Sec translocon directly [10,13,14], suggesting that in bacteria a single polypeptide is sufficient for the binding of SRP-RNCs to the membrane and for coordinating their transfer to the Sec translocon.

Despite the importance of the FtsY-membrane interaction, in cell fractionation studies about 60% of FtsY were found in the cytosolic cell fraction and only 40% at the membrane [15]. However, the function of the soluble FtsY is still largely enigmatic. Despite its inability to induce SRP release from the RNCs, *in vitro *data have shown that soluble FtsY is able to associate with SRP in the absence of membranes [16-20] and that the presence of RNCs strongly accelerates the FtsY-SRP complex formation in solution [21]. These data are consistent with a model in which FtsY binds to SRP-RNCs already in the cytosol and then targets the SRP-RNC complex to the membrane [22]. Nevertheless, the formation of a soluble FtsY-SRP-RNC complex is probably not a prerequisite for cotranslational targeting in bacteria, because FtsY derivatives which are permanently membrane-tethered via a transmembrane domain are functional *in vivo *[23].

In the current study we have analysed the contribution of soluble FtsY to cotranslational targeting *in vitro *and found that, although FtsY can bind to SRP-RNCs in solution, these soluble FtsY-SRP-RNC complexes were not efficiently targeted to the membrane. Furthermore, we re-examined the localization of FtsY *in vivo *by fluorescence microscopy and found it almost exclusively located at the cytoplasmic membrane.

## Results and discussion

### Soluble FtsY binds to SRP-ribosome nascent chains but is unable to efficiently target them to the membrane

The presence of a predominantly soluble SRP receptor in bacteria has led to the hypothesis that FtsY binds to SRP-RNCs already in the cytosol and then targets the SRP-RNCs to the membrane [22]. Several studies have shown that FtsY is able to interact with SRP or SRP-RNCs in solution, that is, in the absence of membranes [16-21]. However, whether FtsY-SRP-RNC complexes which were assembled in the absence of membranes are efficiently targeted to the membrane, has not so far been analysed.

**Figure 1:**
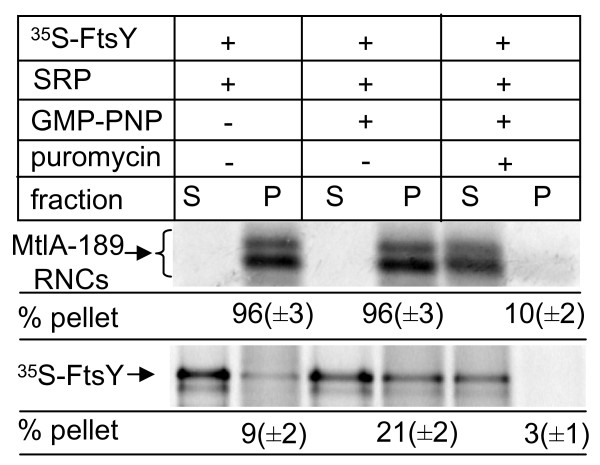
**Binding of FtsY to ribosome nascent chains requires the presence of signal recognition particles (SRP) and guanosine 5'(*β*,-γ imido) triphosphate (GMP)-PNP**. MtlA189 RNCs were *in vitro *synthesized in the presence of purified SRP and *in vitro *synthesized, purified FtsY. When indicated puromycin or GMP-PNP were added. Ribosome-nascent chains (RNCs) were subsequently separated by centrifugation through a sucrose cushion into pellet fraction (P) and supernatant (S), which were analysed on SDS-polyacrylamide gel electrophoresis (SDS-PAGE). Both panels of the figure correspond to the same gel but were separated due to the large size-difference between FtsY and the RNCs. The radioactive material was quantified using a phosphor imager and the Imagequant software. Three independent experiments were performed.

For addressing this crucial question we employed a purified *in vitro *transcription/translation system. In a first approach we analysed whether binding of FtsY to RNCs in the absence of membranes was also observed in this *in vitro *system. RNCs of the SRP-dependent membrane protein mannitol permease (MtlA) were *in vitro *synthesized in the presence of SRP and incubated with either purified *in vitro *synthesized FtsY or a buffer. RNCs were then isolated by centrifugation through a sucrose cushion and the supernatant (containing unbound FtsY) and the pellet fractions (containing RNCs and bound FtsY) were separated on SDS polyacrylamide gel (SDS-PAGE). Phosphor imaging revealed that the 189 amino acid long RNCs of MtlA (MtlA-189 RNCs) were almost exclusively found in the pellet fraction after centrifugation (Figure 1). However, we observed only a weak binding of purified ^35^S-labelled FtsY to these SRP-RNCs, unless the non-hydrolysable GTP-analogue guanosine 5'(*β*,-ν imido) triphosphate (GMP-PNP) was added. This was not the result of a GMP-PNP induced aggregation of FtsY, because, after adding puromycin to dissociate the ribosome, almost 100% of both FtsY and MtlA-189 were found in the supernatant (Figure 1). The addition of GMP-PNP has been shown to stabilize the FtsY-SRP interaction *in vitro *[18]. In agreement with previously published data [18], the binding of FtsY to MtlA-189 RNCs was only observed in the presence of SRP (data not shown). Thus, these *in vitro *data confirm that FtsY cannot bind directly to RNCs but only via an SRP.

**Figure 2:**
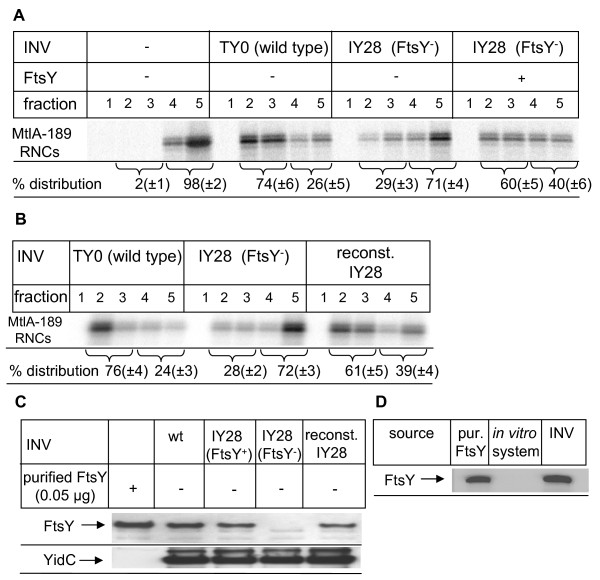
**FtsY-depleted inner membrane vesicles are unable to support co-translational targeting**. (A) MtlA189 RNCs were incubated with wild-type (wt) INV, FtsY-containing IY28 INV (FtsY^+^) and FtsY-depleted IY28 INV (FtsY) (1 μl, 50 μg protein) and subjected to flotation gradient centrifugation. Subsequently, the gradient was separated into five fractions, which were analysed on SDS-PAGE. Fraction 2 and 3 of the gradient correspond to the membrane fraction, while fraction 4 and 5 reflect the ribosome-nascent chains that did not bind to the inner membrane vesicles [INV]. The sum of the radioactive material in all fractions was set as 100% and the amount of radioactive material in the individual fractions was quantified using a phosphor imager. (B) Flotation gradient analyses as in A, but FtsY-depleted IY28 INV were pre-incubated with purified FtsY (2 μg/25 μl; reconst. IY28). (C) Western blot analyses of the INV (5 μl; 250 μg protein) analysed in A and B using polyclonal antibodies against FtsY and against the integral membrane protein YidC. (D) Western blot analyses of the *in vitro *transcription/translation system used in this study using polyclonal anti-FtsY antibodies. Two equivalents of the *in vitro *system components were loaded. One equivalent corresponds to the amount required for *in vitro *protein synthesis. As control wild-type INV (200 μg protein) and purified FtsY (0.1 μg) were loaded.

The ability of FtsY to bind to SRP-RNC complexes in the absence of membranes would support the hypothesis that FtsY binds to its cargo already in the cytosol, and then targets it to the membrane. Flotation gradient analyses have been successfully used to determine the membrane targeting of RNCs, because this approach allows the differentiation between RNCs that were targeted to the SecYEG-containing membrane fractions of the gradient (fractions 2 and 3) and those that were not targeted or aggregated (fractions 4 and 5) [24,25]. MtlA-189 RNCs were *in vitro *synthesized in the presence of purified SRP and subjected to flotation gradient centrifugation. In the absence of inner membrane vesicles (INV), almost 100% of the MtlA-189 RNCs were found in the pellet fraction of the gradient, but in the presence of wild-type INV almost 75% of the RNCs were bound to the membrane (Figure 2A). For analysing the contribution of FtsY to membrane targeting, we employed INV derived from the *E. coli *strain IY28. In this strain, the expression of FtsY is under the control of the arabinose promoter which allows the gradual depletion of FtsY by growing cells without arabinose. In comparison to wild-type INV, targeting of MtlA-189 RNCs to FtsY-depleted IY28 INV was significantly reduced (Figure 2A), but was almost fully restored when purified FtsY, together with the MtlA-189 RNCs, was added to the FtsY-depleted INVs (Figure 2A).

By adding purified FtsY and the RNCs to FtsY-depleted INV, we were unable to distinguish between targeting that involves the formation of soluble FtsY-SRP-RNC complexes and targeting that is achieved because FtsY binds to the membrane first and only then accepts the SRP-RNCs. We therefore incubated FtsY-depleted IY28 INV with purified FtsY and re-isolated these reconstituted INV by centrifugation before adding the MtlA-189 RNCs. Flotation gradient analyses revealed significant targeting of MtlA-189 RNCs to the reconstituted IY 28 INV (Figure 2B).

Western blotting confirmed that FtsY was almost undetectable in FtsY-depleted INV (Figure 2C) and revealed that the FtsY content of the reconstituted IY28 INV was comparable to that of wild-type INV (Figure 2C). Antibodies against the membrane protein YidC were used as a control in order to ensure that comparable amounts of protein were loaded (Figure 2C). As the *in vitro *transcription/translation system used in these experiments does not contain soluble FtsY ([26], Figure 2D), these data demonstrate that the formation of a soluble FtsY-SRP-RNC complex is obviously not essential for efficient targeting.

**Figure 3:**
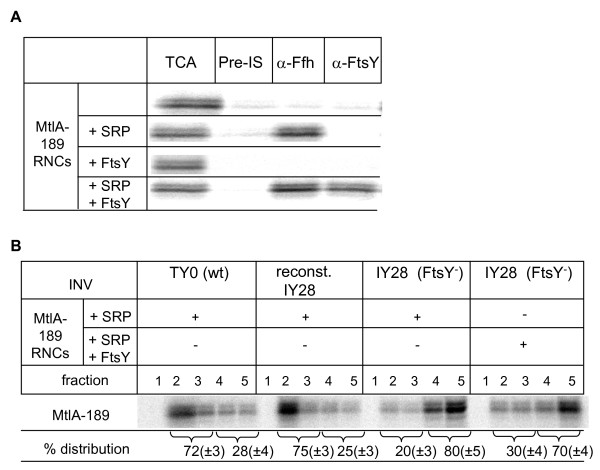
**Soluble FtsY- signal recognition particle (SRP) - MtlA189 ribosome-nascent chains (RNCs) are not efficiently targeted to inner membrane vesicles**. (A) *In vitro *synthesized MtlA189 RNCs were incubated with purified SRP, FtsY or both in the presence of guanosine 5'(*β*,-γ imido) triphosphate (GMP)-PNP. The RNCs were subsequently isolated by centrifugation through a sucrose cushion and resuspended in buffer (50 mM triethanolamine acetate, pH 8; 50 mM potassium acetate; 5 mM magnesium acetate; 1 mM DTT). One volume was directly trichloroacetic acid precipitated and five volumes were subjected to immune precipitation using sepharose-bound polyclonal antibodies against either Ffh or FtsY. As control pre-immune serum (Pre-IS) was used. (B) The MtlA189 RNCs shown in A, and containing either SRP or SRP and FtsY, were incubated with the indicated inner membrane vesicles (1 μl, 50 μg protein) and subjected to flotation gradient centrifugation.

Although the co-sedimentation assays (Figure 1) indicated that FtsY can form stable complexes with SRP-RNCs in the presence of GMP-PNP, it is currently unknown whether these soluble FtsY-SRP-RNC complexes are targeted to the membrane. We, therefore, *in vitro *synthesized MtlA-189 RNCs in the presence of SRP and subsequently incubated these SRP-RNCs with purified FtsY in the presence of GMP-PNP. The non-bound FtsY was then removed by centrifugation of the RNCs through a sucrose cushion and binding of FtsY to the SRP-RNCs was analysed by immune precipitation. MtlA-189 RNCs that were synthesized in the absence of SRP were precipitated by trichloroacetic acid (TCA) but not by antibodies against Ffh or FtsY (Figure 3A). If the synthesis was performed in the presence of SRP, α-Ffh antibodies, but not α-FtsY or pre-immune serum (Pre-IS), precipitated the MtlA189 RNCs, demonstrating that SRP was bound to these RNCs. When the SRP-MtlA189-RNCs were incubated with FtsY, immune precipitation was also achieved with α-FtsY antibodies, indicating the formation of FtsY-SRP-MtlA-189 RNC complexes in solution. In the absence of SRP, FtsY was unable to bind to RNCs, which, again, demonstrates that FtsY obviously does not bind directly to RNCs but only via SRP.

The targeting of the SRP-MtlA-189 RNC complexes and the FtsY-SRP-MtlA-189 RNC complexes was then analysed using flotation gradient analyses. Efficient targeting of SRP-MtlA-189 RNCs was observed only when membrane-bound FtsY was present - for example, in the presence of wild-type INV or FtsY-reconstituted IY28 INV - but not with FtsY-depleted IY28 INV (Figure 3B). Strikingly, we did not observe efficient targeting of soluble FtsY-SRP-MtlA-189 RNCs complexes to FtsY-depleted IY28 INV (Figure 3B). Although a complex formation between FtsY and SRP-RNCs is possible in solution, this early interaction obviously does not permit the subsequent membrane targeting. It is important to emphasize that the addition of GMP-PNP does not prevent targeting, because GTP hydrolysis is not required for the insertion of the signal peptide into the translocon but rather for the dissociation of the SRP-SR complex [20].

### FtsY is predominantly membrane-localized *in vivo*

**Figure 4:**
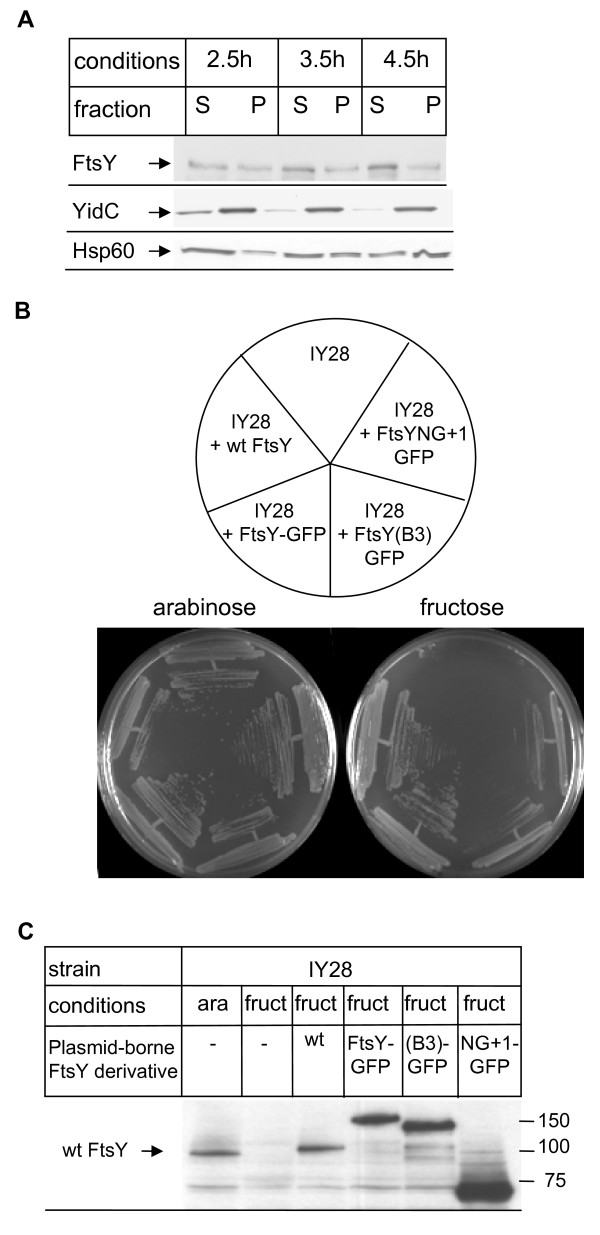
**Cellular localization of FtsY *in vitro *and *in vivo***. (A) *Escherichia coli *cells were grown on LB medium up to mid-exponential phase (OD_600 _1.2). Cell breakage was performed using a French pressure cell in the presence of protease inhibitors. Unbroken cells and large cell fragments were removed by centrifugation and the supernatant of this centrifugation was then separated by ultracentrifugation into the soluble fraction (S) and the pellet fraction (P). Conditions indicate the centrifugation time in a Ti50.2 rotor at 45,000 rpm. After western transfer, the different fractions were analysed using antibodies against FtsY, against the integral membrane protein YidC and against the soluble protein Hsp60 (GroEL). (B) The functionality of the FtsY-green fluorescent protein (GFP) constructs was analysed by expressing plasmid-borne copies in the conditional FtsY depletion strain IY28. IY 28 containing either no plasmid (IY28) or the indicated plasmids was grown on LB-plates in the presence or absence of arabinose. Wt FtsY corresponds to untagged FtsY, wt FtsY-GFP corresponds to full length FtsY fused C-terminally to GFP. FtsY(B3)-GFP and FtsY(NG+1)-GFP correspond to GFP-tagged FtsY mutants which exhibit reduced activity due to impaired membrane binding. The growth experiments were performed in the absence of IPTG for preventing high-level expression of the plasmid-borne FtsY derivatives. (C) Western blot analyses of IY28 cells containing either no or the indicated plasmids. Cells were grown in the presence of arabinose or fructose but in the absence of IPTG.

The inability of soluble FtsY to induce targeting is puzzling in light of biochemical data which have localized more than 60% of FtsY to the cytosol in *E. coli *[15]. This latter study used freezing/thawing cycles combined with ultrasonic treatment for cell breakage. In order to analyse whether the distribution of FtsY was influenced by the method used for cell breakage, we employed a different technique - a French pressure cell. Using the standard protocol, at least 50% of FtsY were present in the cytosolic fraction (Figure 4A). However, under these conditions a significant portion of the integral membrane protein YidC was also still detectable in the supernatant. Using prolonged centrifugation times (up to 4.5 h) led to the almost complete sedimentation of YidC and also to a significant sedimentation of the soluble protein Hsp60. In contrast, even under these conditions, FtsY was largely present in the supernatant (Figure 4a).

The *in vitro *data demonstrated that soluble FtsY does not contribute significantly to co-translational targeting and so it is possible that soluble FtsY is involved in processes other than SRP-dependent targeting. This could also explain the vast excess of FtsY over SRP (10.000 molecules FtsY versus 100 molecules Ffh/*E. coli *cell) [27]. Alternatively, because FtsY is only peripherally attached to the membrane [8,11], it is also conceivable that during cell breakage a significant fraction of FtsY is detached from the membrane. We therefore analysed the distribution of FtsY in *E. coli *by non-invasive fluorescence microscopy using an FtsY derivative with a C-terminally fused GFP-(green fluorescent protein) tag. The functionality of this construct was confirmed by testing its ability to suppress the growth defect of IY28 cells. These cells, carrying only the arabinose-inducible endogenous *ftsY *gene, are unable to grow on fructose-containing plates (Figure 4B). However, expressing plasmid-borne copies of wild-type FtsY, or of the FtsY-GFP construct, *in trans *restored growth completely (Figure 4B). As a control, we also fused GFP to the C-terminus of the FtsY mutants FtsY(B3) and FtsY(NG+1). Both FtsY derivatives carry mutations/deletions in the lipid-binding domains of FtsY and show reduced membrane binding [9,28]. As a result, they are only partially active and were unable to completely restore the growth of IY28 on fructose media, despite their higher expression level in comparison to endogenous FtsY (Figure 4C)

**Figure 5:**
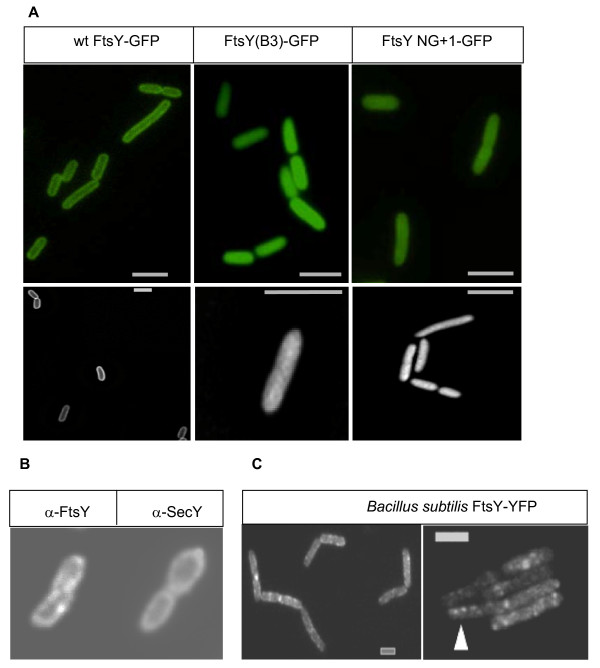
**FtsY is predominantly membrane bound *in vivo***. (A) Wild-type *Escherichia coli *cells carrying different FtsY-green fluorescent protein derivatives as described in Figure. 4 were analysed by fluorescence microscopy. The upper panel displays the original image and the lower panel a processed image after a three dimensional deconvolution of Z-stacks. (B) The localization of FtsY and the integral membrane protein SecY were analysed in wild-type *E. coli *cells containing only the endogenous amounts of FtsY and SecY. For this purpose, immune-fluorescence was carried out using polyclonal SecY and FtsY antibodies and Alexa fluor 555 labelled secondary antibodies. (C) *Bacillus subtilis *cells expressing FtsY-YFP as sole source of the protein growing exponentially; foci along the lateral cell membrane are indicated by white triangle. White bar 2 μm.

When FtsY-GFP was expressed in wild-type *E. coli *and analysed by fluorescence microscopy, it was predominantly localized to the membrane (Figure 5A). A three dimensional de-convolution of Z-stacks of FtsY-GFP confirmed that there is little to no FtsY within the cytosol of E. coli. Quantifying the fluorescent signals revealed that more than 80% of FtsY were associated with the cell membrane (Figure 5A, Additional file 1). Considering that, even after de-convolution, the signal in the cytosol is influenced by the fluorescence of the distal and proximal membranes, the amount of membrane-bound FtsY is most likely to be even higher. In order to exclude the possibility that the presence of the GFP-tag favoured the exclusive membrane localization of FtsY, we analysed the localization of FtsY(NG+1)-GFP and FtsY(B3)-GFP. These FtsY derivatives were uniformly distributed throughout the whole *E. coli *cell, - that is, they were present both at the membrane and in the cytosol - which is in agreement with their reduced ability to bind to the *E. coli *membrane [9,28] (Figure 5A, Additional files 2 and 3). Although we considered it unlikely that the predominant membrane localization of FtsY was the result of the higher expression-level of the GFP-constructs in comparison to the endogenous FtsY (Fig. 4C), we also analysed the localization of the endogenous FtsY in wild-type *E. coli *cells by immune-fluorescence, using fluorescently labelled antibodies against α-FtsY IgGs. This approach confirmed that the endogenous, non-tagged FtsY was also almost exclusively localized to the inner membrane of *E. coli *(Figure 5B). The localization of FtsY was not significantly different from the localization of the integral membrane protein SecY (Figure 5B).

In *E. coli *and other proteobacteria, membrane localization of FtsY is mainly achieved via two cooperating lipid binding domains; in Gram positive bacteria and archaea, FtsY contains either a true transmembrane helix or a single, but extended, lipid-binding helix [29-31]. We therefore analysed the localization of FtsY also in the Gram positive bacterium *Bacillus subtilis *by creating a C-terminal YFP (yellow fluorescent protein) fusion. *B. subtilis *cells expressing only the FtsY-YFP fusion from the original gene locus were viable, indicating that FtsY-YFP was a functional substitute of the essential endogenous FtsY [29]. Unlike *E. coli *FtsY, *B. subtilis *FtsY formed defined foci along the lateral cell membrane (Figure 5C, indicated by white triangle), and occasionally at the cell poles (in 20% of the cells showing defined signals). We did not detect FtsY foci in about 25% of all cells, probably because they were too faint. Fluorescence within the cytosol was similar to background fluorescence in cells lacking any YFP fusion, showing that, despite the differences in the mode of membrane binding between *E. coli *FtsY and *B. subtilis *FtsY, the latter is also highly enriched at the cytoplasmic membrane (Figure 5C).

So far, the function of soluble FtsY in *E. coli *and other prokaryotes has been mysterious, and our data now demonstrate that soluble FtsY represents only a minor species *in vivo*. The high amount of soluble FtsY observed after cell fractionation is most likely to be an artefact of cell breakage. Thus, the exclusive membrane localization of FtsY/SR*α *appears to be an evolutionary conserved feature, although the exact mechanisms of tethering FtsY/SR*α *to the membrane are different. In eukaryotes, it is mainly achieved via the protein-protein contact to SR*β*, while in prokaryotes, protein-lipid contact is dominating [8,9,11]. These differences in the mode of membrane binding also explain why only the SRP- and GTP-interacting NG domains of FtsY/SR*α *are highly conserved, while the low conserved N-terminal domains are adapted to their particular mode of membrane binding.

The predominant membrane localization of FtsY in bacteria also mitigates an otherwise crucial obstacle to the bacterial SRP cycle: if SRP and FtsY interacted efficiently with each other in the cytosol, they would probably undergo futile cycles of GTP hydrolysis. To prevent this, FtsY-SRP complex formation or stability should respond to cargo loading. Kinetic studies have demonstrated that the formation of the SRP-SR complex is slow in the absence of RNCs [4,21,32,33]. However, even in the presence of cargo, significant GTP hydrolysis and complex dissociation is observed in the solution [21]. Thus, to prevent futile GTP hydrolysis, the GTPase cycle should also respond to the presence of membranes. A recent study has shown that mutating the lipid binding domain in chloroplast FtsY results in the loss of membrane-induced GTPase stimulation and higher rates of GTP hydrolysis in solution [34]; a similar link between GTP hydrolysis and membrane binding has also been proposed in *E. coli *[35].

Biochemical data have shown that FtsY binds preferentially to negatively charged phospholipids [8,11,35] and at least transiently to the SecY translocon [10,13,14]. Due the limited number of SecYEG translocons (300-500/*E. coli *cell, [27]), the exclusive membrane localization of FtsY suggests that *in vivo *FtsY is mainly bound to phospholipids. However, this probably excludes phospholipids as major player in regulating co-translational protein targeting in bacteria. Instead, the FtsY-translocon contact might be the key element in controlling the GTPase cycle and the insertion of the substrate into the translocation channel. In eukaryotes, the GTPase domain of the SR*β *subunit appears to be crucial for the coordination of RNC binding with the subsequent transfer to the Sec translocon [4,6,7,36]. As FtsY can directly interact with the SecYEG translocon [10,13,14], the SRP receptor in prokaryotes can probably mediate both functions: binding of the SRP-RNCs and coordinating their transfer to the Sec translocon.

So far, the kinetic analyses of the bacterial SRP cycle have only been performed in the absence of membranes and, in some cases, with N-terminal truncated FtsY derivatives lacking at least the first lipid-binding domain [20,21,37,38]. Although these studies have provided a wealth of important information about the SRP cycle, it is essential to determine the impact of membranes and the Sec translocon on the kinetics of the SRP cycle in future studies. Finally, our data also show that, although FtsY can bind to SRP-RNCs, in solution it fails to efficiently target these SRP-RNCs to the membrane. It seems likely that the interaction of FtsY with SRP-RNCs in solution interferes with the subsequent binding of FtsY to lipids or SecY, although this needs to be confirmed. Nevertheless, considering that soluble FtsY obviously represents only a minor species in *E. coli*, its low targeting activity should not impose any problems for the cell.

## Conclusion

The prokaryotic SRP receptor lacks a membrane-integral SR*β *subunit and has been mainly localized to the cytosolic cell fraction after cell breakage. This predominant cytosolic localization has been taken as an indication that, in bacteria, the SRP receptor interacts with the SRP-RNCs already in the cytosol and then targets them to the membrane. Our *in vivo *data now show that, despite the lack of a SR*β *subunit, FtsY is almost exclusively membrane bound in bacteria and that targeting of SRP-RNCs is only possible when FtsY is membrane-bound. Thus, the exclusive membrane localization of FtsY/SR*α *appears to be an evolutionary conserved feature, although the exact mechanisms of tethering FtsY/SR*α *to the membrane are different between prokaryotes and eukaryotes.

## Methods

### Strains, plasmids, cell growth and fractionation methods

The following *E. coli *strains were used: Bl21, MRE 600 [39], TY0 [40] and IY28 (obtained from Eitan Bibi). The conditional FtsY-depletion strain IY28 was routinely grown on LB medium supplemented with 0.4% arabinose. For FtsY-depletion, IY28 was grown overnight on media supplemented with 0.2% arabinose. After harvesting the cells by centrifugation, cells were washed twice with media lacking arabinose and used to inoculate cultures containing either 0.2% arabinose to induce FtsY expression or 0.2% fructose for FtsY depletion. Growth was monitored by measuring the optical density at 600 nm. For *in vitro *protein synthesis the following plasmids were used: pKSM717-MtlA (MtlA) [26], pTP37 (FtsY) [41] and pET19b-Ffh [42]. For FtsY-GFP fusions, FtsY was PCR amplified using pTP37 as template and ligated into pTrc99a using NcoI and BamHI restriction, resulting in plasmid pTrc99a-FtsY. pTrc99a-FtsY was then digested with HindIII and the100 bp HindIII fragment (containing the C-terminal His Tag and the stop codon of FtsY) was replaced by a 754 bp long polymerase chain reaction (PCR) product containing the GFP. This PCR product was generated by using the plasmid pCA24NCycY-GFP (National Bioresource Project Japan) and the primer FtsY-GFPfw (5'-GAAACAGTCAAGCTTC-GCGGCC-3') and FtsY-GFPrev (5'-CCTGCAGCCA-AGCTTAATTAGCT-3'). The construction of the FtY(B3) and FtsY NG+1 GFP fusions followed the same strategy, with the exception that the first PCR was performed with pSAFtsY(NG+1) [14] or pTP37-FtsYB3 [9] as templates.

In order to generate an FtsY-YFP fusion in *B. subtilis*, the last 500 bp of the *ftsY *gene were amplified by PCR and were cloned into plasmid pSG1164y [43] using *Apa*I and *Eco*RI restriction sites. *B. subtilis *PY79 was transformed with the resulting plasmid, selecting for chloramphenicol resistance, leading to an integration of the plasmid into the chromosome at the *ftsY *locus. All chemicals and growth media components were obtained from Roth (Karlsruhe, Germany) unless otherwise stated.

For cell fractionation, *E. coli *cells were grown in INV medium at 37°C up to an OD_600 _of 1.2. After harvesting, the cells were resuspended in buffer A (50 mM triethanolamine acetate, pH 7.5, 0.25 M sucrose, 1 mM EDTA, 1 mM DTT, 5 ml/1 g cells) and broken by three passages through a French Pressure Cell at 8000 psi in the presence of phenylmethylsulfonyl fluoride (1 mM) and Complete-EDTA-free protease inhibitor (Roche, Mannheim, Germany). Unbroken cells and large cell debris were removed by centrifugation for 30 min at 5000 rpm in a SS34 rotor (Kendro-Sorvall, Langenselbold, Germany). The supernatant was then subjected to an ultracentrifugation step to separate the membrane fraction from the soluble fraction. The standard protocol included a centrifugation at 45,000 rpm in a Ti50.2 rotor (Beckman-Coulter, Krefeld, Germany) for 2.5 h at 4°C. The membrane pellet was resuspended in INV buffer (buffer A without EDTA).

### *In vitro *synthesis

The *in vitro *synthesis of RNCs and flotation gradient analyses of membrane-bound RNCs were performed (as described in [24-26]). For flotation gradients, 1 μl of INV (50 μg protein) were incubated with a 50 μl *in vitro *reaction mixture. For sedimentation assays, 50 μl RNCs were pelleted through a sucrose cushion (100 μl, 40 mM triethanolamine acetate, pH 7.5; 70 mM potassium acetate; 10 mM magnesium acetate, 1 mM DTT and 580 mM sucrose) for 60 min at 90,000 rpm in a Beckmann 100.2 rotor. *In vitro *synthesized FtsY and Ffh were purified via Talon metal affinity resin (as described in [14]) before incubation with RNCs. SRP was reconstituted by incubating purified Ffh and purified 4.5S RNA in a 1:2 ratio for 15 min at 25°C [37]. For reconstituting SRP with *in vitro *synthesized Ffh, ^35^S-labelled Ffh was incubated with 1 μg of 4.5S RNA as described above and then purified via Talon metal affinity resin. FtsY, Ffh and 4.5S RNA were purified (as described in [24,42]). Immune precipitation was performed in fivefold scaled-up reactions using polyclonal rabbit antibodies against FtsY and Ffh, covalently linked to protein A-sepharose matrix [24]. Radioactively labelled proteins were separated on 13% SDS-polyacrylamide gels and visualized using a phosphorimager. The radioactive material was quantified using the Imagequant software (GE Healthcare, Munich, Germany).

### Fluorescence microscopy and immune fluorescence

In order to investigate GFP-tagged proteins, bacterial cells were immobilized on a microscope slide with low melting agarose. For immune fluorescence, *E. coli *cells were grown to exponential phase (OD_600_~0.8 to 1.2) in LB medium supplemented with the appropriate antibiotics and 0.4% arabinose. Five hundred microlitres of culture were fixed with 100 μl 16% paraformaldehyde in phosphate buffered saline (PBS, 10 mM Na_2_HPO_4_/NaH_2_PO_4_, 150 mM NaCl, 3 mM KCl, pH 7.4) with 0.125% glutardialdehyde for 15 min at room temperature and afterwards for an additional 30 min on ice. Following three washing steps with PBS, cells were resuspended in 500 μl GTE buffer (50 mM glucose, 10 mM EDTA, 20 mM Tris pH 7.5). Lysozyme was added to a final concentration of 2 μg/ml and an appropriate amount of the cell suspension was directly applied to the wells of an eight-well microscope slide (Thermo Scientific, Braunschweig, Germany) coated with Poly-L-Lysine (Sigma Aldrich, Steinheim; Germany). After 5 min of incubation, the excess cells were removed using a vacuum pump and the wells were washed once with PBS. The samples were then blocked with 2% bovine serum albumine in PBS for 15 min at room temperature. Primary antibodies (rabbit-raised polyclonal anti-FtsY or anti-SecY anti-serum) in 2% BSA/PBS were added directly afterwards and incubated over night at 4°C. The dilution yielding the best results was determined by titration for each antibody independently. The next-day samples were washed 10 times with PBS and, subsequently, an Alexa Fluor 555 coupled goat anti-rabbit antibody (Invitrogen; Karlsruhe, Germany) was added at a final concentration of 4 μg/ml in 2% BSA/PBS. After incubation for 90 min in the dark at room temperature and washing with PBS, the samples were dried, an appropriate amount of SlowFade Gold (Invitrogen, Karlsruhe, Germany) was applied and the slide was covered with microscope cover slips. Stained proteins were visualized using an Olympus BX-51 fluorescence microscope at a 100× magnification with a numerical aperture of 1.4 and a Cy3 fluorescence filter set. Images were acquired with a charge-coupled device camera (F-View, Olympus, Hamburg, Germany). Z-stacks were captured on a Zeiss Axioimager Microscope with an objective piezo. A three dimensional deconvolution was performed using Autodeblur X software.

## Abbreviations

GFP: green fluorescent protein; GMP-PNP: guanosine 5'(*β*,-γ imido) triphosphate; INV: inner membrane vesicles; MtlA: mannitol permease; PAGE: polyacrylamide gel electrophoresis; PBS: phosphate buffered saline; PCR: polymerase chain reaction; Pre-IS: pre-immune serum; RNC: ribosome-nascent chain; SR: signal recognition particle receptor; SRP: signal recognition particle; TCA: trichloroacetic acid; YFP: yellow fluorescent protein.

## Authors' contributions

MM carried out the *in vitro *studies and the cell fractionation experiments. DB and BW constructed and analysed the GFP-tagged FtsY derivatives. FH and PG analysed the localization of *B. subtilis *FtsY. HGK participated in the *in vitro *studies and drafted the manuscript. All authors read and approved the final manuscript.

## Supplementary Material

Additional file 1 **Movie S1 - Membrane localization of green fluorescent protein (GFP)-tagged wild type FtsY**. GFP-tagged wild type FtsY was expressed from a plasmid in DH5α and localization was monitored on a motorised Zeiss Axioobserver microscope using a Cascade EM-CCD camera. Z stacks were acquired with 0.1 μm spacing and were three dimensionally de-convoluted using Autodeblur software. Movies were created in Metamorph and can be displayed with Quicktime movie player. Click here for file

Additional file 2 **Movie S2 - Membrane localization of green fluorescent protein (GFP)-tagged FtsY(B3) mutant derivative**. The GFP-tagged FtsY(B3) mutant derivative was expressed from a plasmid in DH5α and localization was monitored on a motorised Zeiss Axioobserver microscope using a Cascade EM-CCD camera. The FtsY(B3) derivative exhibits reduced activity due to a triple-point mutation within the first lipid-binding domain of FtsY. Z stacks were acquired with 0.1 μm spacing and were three dimensionally de-convoluted using Autodeblur software. Movies were created in Metamorph and can be displayed with Quicktime movie player. Click here for file

Additional file 3 **Movie S3 - Membrane localization of green fluorescent protein (GFP)-tagged FtsY(NG-1) mutant derivative**. The GFP-tagged FtsY(NG+1) derivative was expressed from a plasmid in DH5α and localization was monitored on a motorised Zeiss Axioobserver microscope using a Cascade EM-CCD camera. The FtsY(NG+1) is an N-terminally truncated FtsY-derivative, which contains only one out of two lipid binding domains and displays reduced activity. Z stacks were acquired with 0.1 μm spacing and were three dimensionally de-convoluted using Autodeblur software. Movies were created in Metamorph and can be displayed with Quicktime movie player. Click here for file
